# Supplementary material for: Neighborhood Characteristics and the Mental Health of Caregivers Cohabiting with Care Recipients Diagnosed with Alzheimer’s Disease
Source: Int J Environ Res Public Health. 2021 Jan 21;18(3):913. doi: 10.3390/ijerph18030913 (PMC7908545; doi:10.3390/ijerph18030913)
Supplement: Supplementary file 1 [file ijerph-18-00913-s001.pdf]

Supplemental Table 1. Comparing caregiver demographics between those in the study sample vs. the full sample, 2010.

Caregiver Demographics	Cohabited Caregivers from Study Sample (*n* = 212)	All Cohabited Caregivers from the Registry Subsample (*n* = 224)
	N **(Percentage)**	***N*** **(Percentage)**
CESD score, mean (S.D.)	10.2 ± 6.31	10.2 ± 6.4
ZBI score, mean (S.D.)	5.9 ± 3.9	5.8 ± 3.9
NPI-Q-CD, mean (S.D.)	12.1 ± 10.4	12.3 ± 10.6
Age, mean (S.D.)	58.9 ± 11.1	58.8 ± 10.2
Sex/Gender		
Male	31 (14.6)	33 (14.8)
Female	181 (85.3)	191 (85.2)
Race/Ethnicity		
Non-Hispanic Black	117 (55.1)	124 (55.1)
Other [a]	95 (44.8)	100 (44.8)
Employment		
Employed	70 (33.0)	72 (32.2)
Retired/Unemployed	113 (53.3)	120 (53.9)
Other [b]	29 (13.6)	32 (13.9)
Relationship to the Care Recipient		
Spouse	35 (16.5)	36 (16.1)
Child	144 (67.9)	152 (67.7)
Other [c]	33 (15.5)	36 (16.1)

[a] "Other" race/ethnicity included non-Hispanic white, Hispanic/Latinx, and Asian.

[b] Retired and unemployed included fully retired, unemployed, and homemaker, and employed included employed full time, employed part time, and retired but working part time.

[c] "Other" relationship to care recipient included daughter-in-law, sister, brother, grandchild, niece or nephew, and other.

NPI-Q-CD: Neuropsychiatric Inventory Questionnaire- Caregiver Distress, CESD: Center for Epidemiologic Studies Depression Scale, ZBI: Zarit Burden Interview.

Supplemental Table 2. Instruments used to measure the caregivers' mental health outcomes.

Instrument	0	1	2	3	4	5	Range
Center for Epidemiologic Studies Depression Scale–Revised	Rarely/ None of the time	Some of the time	Occasionally	Most of the time	n/a	n/a	0–30
Zarit Burden Interview	Never	Rarely	Sometimes	Quite frequently	Nearly always	n/a	0–16
Neuro-Psychiatric Inventory Questionnaire Caregiver Distress	Not distressing at all	Minimal	Mild	Moderate	Severe	Extreme or very severe	0–60

Supplemental Table 3. Mean scores of the NPI-Q domains in care recipients with Alzheimer's disease, 2010 (n = 212). [a]

NPI Domain	Mean ± S.D. [b]
NPI total score	26.3 ± 22.3
Delusions	1.8 ± 3.1
Hallucinations	1.9 ± 3.1
Agitation/aggression	3.1 ± 3.5
Depression/dysphoria	2.3 ± 3.5
Anxiety	1.7 ± 3.1
Euphoria/elation	0.7 ± 1.7
Apathy	2.8 ± 3.7
Disinhibition	1.3 ± 2.7
Irritability	2.8 ± 3.7
Motor disturbances	2.8 ± 3.7
Sleep and nighttime disturbances	2.7 ± 3.8
Appetite/eating change	1.9 ± 3.3

[a] NPI-Q is the Neuropsychiatric Inventory Questionnaire that assesses neuropsychiatric symptoms.

[b] S.D.: standard deviation.

Supplemental Table 4. Unadjusted and adjusted prevalence ratios (PRs) of caregiver depressive symptoms by neighborhood characteristics as defined at the 3-mile buffer, 2010.

Variable	Severe Neuropsychiatric Symptoms (*n* = 112) [c]		Non-Severe Neuropsychiatric Symptoms (*n* = 100) [c]	
	**Unadjusted PR (95% CI)**	**Adjusted PR [d] (95% CI)**	**Unadjusted PR (95% CI)**	**Adjusted PR [d] (95% CI)**
Rurality [a]				
Rural	1.05 (0.83–1.35)	0.98 (0.75–1.27)	0.88 (0.65–1.22)	0.98 (0.69–1.39)
Small urban	0.89 (0.66–1.19)	0.96 (0.71–1.28)	0.89 (0.62–1.28)	1.02 (0.68–1.51)
Large urban	1.00 [e]	1.00 [e]	1.00 [e]	1.00 [e]
Percent moved 1 year ago	1.03 (0.95–1.12)	1.04 (0.95–1.12)	0.98 (0.89–1.09)	0.98 (0.89–1.09)
Median household income [b]				
Low	1.18 (0.91–1.53)	1.33 (1.00–1.78)	0.82 (0.59–1.14)	0.80 (0.56–1.17)
Medium	1.19 (0.93–1.54)	1.25 (0.97–1.61)	0.74 (0.53–1.05)	0.77 (0.53–1.12)
High	1.00 [e]	1.00 [e]	1.00 [e]	1.00 [e]

[a] Rurality was measured based on the RUCA (Rural-Urban Commuting Area codes). A "large urban" area was defined as a metropolitan area; a "small urban" area was defined as a metropolitan area with high commuting or a metropolitan area with low commuting; a "rural" area was defined as a micropolitan area core, micropolitan area with high commuting, micropolitan area with low commuting, small town core, small town with high commuting, small town with low commuting, or rural areas.

[b] Median household income categories were based on tertiles, where "low" was <$34,352, "medium" was $34,352–41,927, and "high" was >$41,927.

[c] Severe neuropsychiatric symptoms among those with AD were defined as >median.

[d] Model was adjusted for caregiver age, sex/gender, race/ethnicity, employment, relationship to the care recipient, and sandwich caregiver status.

[e] Reference category.

Supplemental Table 5. Unadjusted and adjusted prevalence ratios of caregiver burden by neighborhood characteristics as defined at the 3-mile buffer, 2010.

Variable	Severe Neuropsychiatric Symptoms (*n* = 112) [c]		Non-Severe Neuropsychiatric Symptoms (*n* = 100) [c]	
	**Unadjusted PR (95% CI)**	**Adjusted PR [d] (95% CI)**	**Unadjusted PR (95% CI)**	**Adjusted PR [d] (95% CI)**
Rurality [a]				
Rural	0.87 (0.67–1.12)	0.87 (0.65–1.17)	0.89 (0.60–1.32)	0.99 (0.65–1.51)
Small urban	0.93 (0.68–1.25)	1.02 (0.74–1.40)	0.86 (0.55–1.36)	0.94 (0.59–1.50)
Large urban	1.00 [e]	1.00 [e]	1.00 [e]	1.00 [e]
Percent moved 1 year ago	1.03 (0.95–1.11)	1.02 (0.94–1.11)	0.96 (0.85–1.09)	0.95 (0.83–1.07)
Median household income [b]				
Low	0.91 (0.70–1.19)	1.05 (0.77–1.33)	0.88 (0.59–1.33)	0.90 (0.57–1.44)
Medium	0.94 (0.72–1.22)	1.01 (0.77–1.33)	0.65 (0.42–1.00)	0.74 (0.47–1.17)
High	1.00 [e]	1.00 [e]	1.00 [e]	1.00 [e]

[a] Rurality was measured based on the RUCA (Rural-Urban Commuting Area codes). A "large urban" area was defined as a metropolitan area; a "small urban" area was defined as a metropolitan area with high commuting or a metropolitan area with low commuting; a "rural" area was defined as a micropolitan area core, micropolitan area with high commuting, micropolitan area with low commuting, small town core, small town with high commuting, small town with low commuting, or rural areas.

[b] Median household income categories were based on tertiles, where "low" was <$34,352, "medium" was $34,352–41,927, and "high" was >$41,927.

[c] Severe neuropsychiatric symptoms among those with AD were defined as >median.

[d] Model was adjusted for caregiver age, sex/gender, race/ethnicity, employment, relationship to the care recipient, and sandwich caregiver status.

[e] Reference category.

Supplemental Table 6. Unadjusted and adjusted prevalence ratios of caregiver distress by neighborhood characteristics as defined at the 3-mile buffer, 2010.

Variable	Severe Neuropsychiatric Symptoms (*n* = 112) [c]		Non-Severe Neuropsychiatric Symptoms (*n* = 100) [c]	
	**Unadjusted PR (95% CI)**	**Adjusted PR [d] (95% CI)**	**Unadjusted PR (95% CI)**	**Adjusted PR [d] (95% CI)**
Rurality [a]				
Rural	0.87 (0.67–1.12)	0.76 (0.61–0.95)	0.89 (0.59–1.32)	0.53 (0.29–1.01)
Small urban	0.93 (0.68–1.25)	0.89 (0.70–1.15)	0.87 (0.55–1.36)	0.63 (0.31–1.27)
Large urban	1.00 [e]	1.00 [e]	1.00 [e]	1.00 [e]
Percent moved 1 year ago	1.02 (0.95–1.11)	0.99 (0.92–1.05)	0.96 (0.85–1.09)	0.93 (0.78–1.11)
Median household income [b]				
Low	0.91 (0.69–1.19)	**1.61 (1.26–2.04)**	0.88 (0.59–1.33)	1.50 (0.73–3.08)
Medium	0.94 (0.72–1.22)	**1.45 (1.17–1.78)**	0.65 (0.42–0.99)	1.20 (0.63–2.28)
High	1.00 [e]	1.00 [e]	1.00 [e]	1.00 [e]

[a] Rurality was measured based on the RUCA (Rural-Urban Commuting Area codes). A "large urban" area was defined as a metropolitan area; a "small urban" area was defined as a metropolitan area with high commuting or a metropolitan area with low commuting; a "rural" area was defined as a micropolitan area core, micropolitan area with high commuting, micropolitan area with low commuting, small town core, small town with high commuting, small town with low commuting, or rural areas.

[b] Median household income categories were based on tertiles, where "low" was <$34,352, "medium" was $34,352–41,927, and "high" was >$41,927.

[c] Severe neuropsychiatric symptoms among those with AD was defined as >median.

[d] Model was adjusted for caregiver age, sex/gender, race/ethnicity, employment, relationship to the care recipient, and sandwich caregiver status.

[e] Reference category.

Bolded estimates indicate statistical significance.

Supplemental Table 7. Unadjusted and adjusted prevalence ratios of caregiver depressive symptoms by neighborhood characteristics as defined at the 1-mile buffer, 2010.

**Variable**	**Unadjusted PR (95% CI)**	**Adjusted PR (95% CI)** [d]
Rurality [a]		
Rural	0.96 (0.78–1.17)	0.97 (0.77–1.21)
Small urban	0.86 (0.68–1.09)	0.91 (0.71–1.16)
Large urban	1.00 [c]	1.00 [c]
Percent moved 1 year ago	1.01 (0.94–1.08)	1.00 (0.94–1.07)
Median household income [b]		
Low	0.99 (0.80–1.23)	1.04 (0.83–1.32)
Medium	0.99 (0.79–1.23)	1.04 (0.83–1.30)
High	1.00 [c]	1.00 [c]

[a] Rurality was measured based on the RUCA (Rural-Urban Commuting Area codes). A "large urban" area was defined as a metropolitan area; a "small urban" area was defined as a metropolitan area with high commuting or a metropolitan area with low commuting; a "rural" area was defined as a micropolitan area core, micropolitan area with high commuting, micropolitan area with low commuting, small town core, small town with high commuting, small town with low commuting, or rural areas.

[b] Median household income categories were based on tertiles, where "low" was <$31,000, "medium" was $31,000–40,758, and "high" was >$40,758.

[c] Reference category.

[d] Model was adjusted for caregiver age, sex/gender, race/ethnicity, employment, relationship to the care recipient, and sandwich caregiver status.

Supplemental Table 8. Unadjusted and adjusted prevalence ratios of caregiver burden by neighborhood characteristics as defined at the 1-mile buffer, 2010.

Variable	Unadjusted PR (95% CI)	Adjusted PR (95% CI) [d]
Rurality [a]		
Rural	0.84 (0.67–1.06)	0.89 (0.69–1.15)
Small urban	0.86 (0.65–1.13)	0.93 (0.70–1.22)
Large urban	1.00 [c]	1.00 [c]
Percent moved 1 year ago	1.00 (0.93–1.07)	0.98 (0.91–1.05)
Median household income [b]		
Low	0.88 (0.69–1.12)	1.00 (0.77–1.31)
Medium	0.82 (0.64–1.05)	0.94 (0.73–1.21)
High	1.00 [c]	1.00 [c]

[a] Rurality was measured based on the RUCA (Rural-Urban Commuting Area codes). A "large urban" area was defined as a metropolitan area; a "small urban" area was defined as a metropolitan area with high commuting or a metropolitan area with low commuting; a "rural area" was defined as a micropolitan area core, micropolitan area with high commuting, micropolitan area with low commuting, small town core, small town with high commuting, small town with low commuting, or rural areas.

[b] Median household income categories were based on tertiles, where "low" was <$31,000, "medium" was $31,000–40,758, and "high" was >$40,758.

[c] Reference category.

[d] Model was adjusted for caregiver age, sex/gender, race/ethnicity, employment, relationship to the care recipient, and sandwich caregiver status

Supplemental Table 9. Unadjusted and adjusted prevalence ratios of caregiver distress by neighborhood characteristics as defined at the 1-mile buffer, 2010.

Variable	Unadjusted PR (95% CI)	Adjusted PR (95% CI) [d]
Rurality [a]		
Rural	0.78 (0.57–1.07)	0.65 (0.45–0.94)
Small urban	0.78 (0.53–1.12)	0.71 (0.48–1.03)
Large urban	1.00 [c]	1.00 [c]
Percent moved 1 year ago	0.98 (0.88–1.09)	0.94 (0.84–1.04)
Median household income [b]		
Low	1.14 (0.81–1.60)	**1.55 (1.05–2.28)**
Medium	1.14 (0.81–1.59)	**1.49 (1.05–2.11)**
High	1.00 [c]	1.00 [c]

[a] Rurality was measured based on the RUCA (Rural-Urban Commuting Area codes). A "large urban" area was defined as a metropolitan area; a "small urban" area was defined as a metropolitan area with high commuting or a metropolitan area with low commuting; a "rural" area was defined as a micropolitan area core, micropolitan area with high commuting, micropolitan area with low commuting, small town core, small town with high commuting, small town with low commuting, or rural areas.

[b] Median household income categories were based on tertiles, where "low" was <$31,000, "medium" was $31,000–40,758, and "high" was >$40,758.

[c] Reference category.

[d] Model was adjusted for caregiver age, sex/gender, race/ethnicity, employment, relationship to the care recipient, and sandwich caregiver status.

Bolded estimates indicate statistical significance.

Supplemental Table 10. Unadjusted and adjusted prevalence ratios of caregiver depressive symptoms by neighborhood characteristics defined at the 3-mile buffer, 2010.

Variable	Unadjusted PR (95% CI)	Adjusted PR (95% CI) [d]
Rurality [a]		
Rural	0.96 (0.78–1.17)	0.97 (0.77–1.21)
Small urban	0.86 (0.68–1.09)	0.91 (0.71–1.16)
Large urban	1.00 [c]	1.00 [c]
Percent moved 1 year ago	1.01 (0.94–1.08)	1.00 (0.94–1.07)
Median household income [b]		
Low	0.99 (0.80–1.23)	1.05 (0.83–1.32)
Medium	0.99 (0.79–1.23)	1.04 (0.83–1.30)
High	1.00 [c]	1.00 [c]

[a] Rurality was measured based on the RUCA (Rural-Urban Commuting Area codes). A "large urban" area was defined as a metropolitan area; a "small urban" area was defined as a metropolitan area with high commuting or a metropolitan area with low commuting; a "rural" area was defined as a micropolitan area core, micropolitan area with high commuting, micropolitan area with low commuting, small town core, small town with high commuting, small town with low commuting, or rural areas.

[b] Median household income categories were based on tertiles, where "low" was <$34,352, "medium" was $34,352–41,927, and "high" was >$41,927.

[c] Reference category.

[d] Model was adjusted for caregiver age, sex/gender, race/ethnicity, employment, relationship to the care recipient, and sandwich caregiver status.

Supplemental Table 11. Unadjusted and adjusted prevalence ratios of caregiver burden by neighborhood characteristics as defined at the 3-mile buffer, 2010.

**Variable**	**Unadjusted PR (95% CI)**	**Adjusted PR (95% CI)** [d]
Rurality [a]		
Rural	0.84 (0.67–1.06)	0.89 (0.69–1.15)
Small urban	0.86 (0.65–1.13)	0.93 (0.70–1.22)
Large urban	1.00 [c]	1.00 [c]
Percent moved 1 year ago	0.99 (0.93–1.08)	0.98 (0.91–1.05)
Median household income [b]		
Low	0.88 (0.69–1.12)	1.00 (0.77–1.31)
Medium	0.82 (0.64–1.05)	0.94 (0.73–1.21)
High	1.00 [c]	1.00 [c]

[a] Rurality was measured based on the RUCA (Rural-Urban Commuting Area codes). A "large urban" area was defined as a metropolitan area; a "small urban" area was defined as a metropolitan area with high commuting or a metropolitan area with low commuting; a "rural" area was defined as a micropolitan area core, micropolitan area with high commuting, micropolitan area with low commuting, small town core, small town with high commuting, small town with low commuting, or rural areas.

[b] Median household income categories were based on tertiles, where "low" was <$34,352, "medium" was $34,352–41,927, and "high" was >$41,927.

[c] Reference category.

[d] Model was adjusted for caregiver age, sex/gender, race/ethnicity, employment, relationship to the care recipient, and sandwich caregiver status.

Supplemental Table 12. Unadjusted and adjusted prevalence ratios of caregiver distress by neighborhood characteristics defined at the 3-mile buffer, 2010.

Variable	Unadjusted PR (95% CI)	Adjusted PR (95% CI) [d]
Rurality [a]		
Rural	0.77 (0.57–1.07)	0.65 (0.45–0.94)
Small urban	0.77 (0.53–1.12)	0.71 (0.48–1.03)
Large urban	1.00 [c]	1.00 [c]
Percent moved 1 year ago	0.98 (0.88–1.09)	0.94 (0.84–1.04)
Median household income [b]		
Low	1.14 (0.81–1.59)	**1.55 (1.05–2.28)**
Medium	1.14 (0.81–1.59)	**1.49 (1.05–2.11)**
High	1.00 [c]	1.00 [c]

[a] Rurality was measured based on the RUCA (Rural-Urban Commuting Area codes). A "large urban" area was defined as ametropolitan area; a "small urban" area was defined as a metropolitan area with high commuting or a metropolitan area with low commuting; a "rural" area was defined as a micropolitan area core, micropolitan area with high commuting, micropolitan area with low commuting, small town core, small town with high commuting, small town with low commuting, or rural areas.

[b] Median household income categories were based on tertiles, where "low" was <$34,352, "medium" was $34,352–41,927, and "high" was >$41,927.

[c] Reference category.

[d] Model was adjusted for caregiver age, sex/gender, race/ethnicity, employment, relationship to the care recipient, and sandwich caregiver status.

Bolded estimates indicate statistical significance.

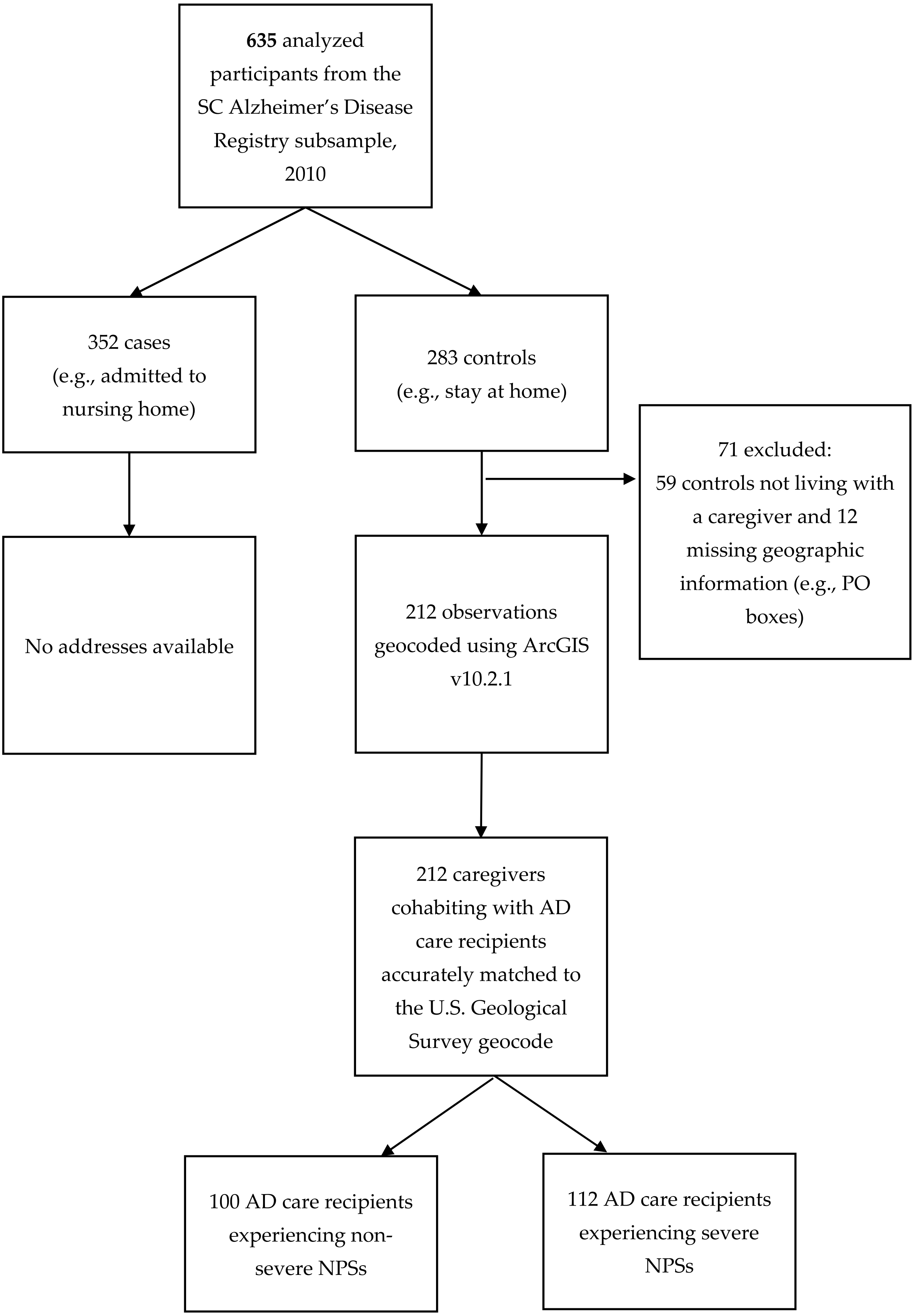

Supplemental Figure 1. Flowchart of the caregiver case selection, 2010. NPS: neuropsychiatric symptom.
